# A Review of the Science Around Monarch Butterflies Should Contain a Complete and Accurate Description of the Science. Comment on Oberhauser, K.S. Eastern North American Monarch Butterfly Conservation Needs and Opportunities: What the Science Tells Us. *Insects* 2026, *17*, 235

**DOI:** 10.3390/insects17070712

**Published:** 2026-07-10

**Authors:** Andrew K. Davis

**Affiliations:** 1 Odum School of Ecology, University of Georgia, Athens, GA 30602, USA; akdavis@uga.edu or adavi749@kennesaw.edu; 2 Department of Ecology, Evolution and Organismal Biology, Kennesaw State University, Kennesaw, GA 30144, USA

Conservation actions should always be based on sound scientific research around the species in question, but more importantly, on the proper interpretation of said research findings. The famous North American monarch butterfly, *Danaus plexippus*, is a species that has been the subject of research for over a century, though in the past 2–3 decades, there has been considerable attention and research directed to their conservation needs, because of observed declines at one stage of their annual cycle, when they overwinter in Mexico. When these winter colony declines were first reported [1], they catalyzed a massive public campaign to help the monarch, and, stimulated much research aimed at understanding why the declines were happening [2,3,4]. However, even when the winter colony declines were first reported, there was opposing evidence from other datasets that did not show evidence of widespread population decline [5], but which was largely dismissed by the authors of the original paper [6]. Unfortunately, that lack of objectivity has been carried forward within certain circles of the monarch research community.

Recently, this journal published an article which purported to be a review of all of the science around the monarch, and which was interpreted as showing how the eastern North American population of monarchs is in peril [7]. As a longtime researcher of monarchs, and one who has been deeply involved in many relevant studies, I was troubled by how this review had reached this conclusion, since there were multiple instances where highly relevant research findings had been mischaracterized, dismissed, or ignored in the article. Below I highlight these instances.

## 1. Breeding Season Censuses

Given that the overall theme of the review was centered around the purported population decline of eastern monarchs, a study that evaluated long-term censuses of monarchs in the summer throughout N. America [8] would therefore be highly relevant. In that study, the analyses of data from 400+ census locations found that there were some regions showing slight increases in monarch abundance, others showed slight decreases, and others showed no change. This included the agriculturally rich Midwestern region; there were decreases and increases in monarch abundance there, but none were statistically significant. Oberhauser discusses this study, but dismissed the findings, because she assumed the data analyses failed to account for the greater abundance of monarchs in the Midwest. This assumption is incorrect, and in fact a subsequent (unpublished) alternative analytical approach of this same dataset revealed that there still was no overall decline in adult monarchs in the Midwest, even when accounting for the greater abundance in that region (T. Meehan, pers. correspondence). Given the continental scope and massive dataset included in that analysis, this conclusion is a key piece of evidence that must be included in any discussion of population status. Not to mention the fact that this same conclusion has been reached by other authors that have examined breeding season census data [9,10].

## 2. Recent Genetic Diversity Study

Great advances have been made in the world of genetics, where nowadays, researchers can examine the genome of a species using archived or contemporary specimens and determine how the level of “genetic diversity” in the species has changed over time, even going back thousands of years. Given that this diversity reflects population size, such analyses are revolutionizing the study of species declines. For example, researchers in Europe determined how populations of honeybees have declined by comparing levels of genetic diversity from archived museum specimens to contemporary ones [11].

A similar approach was recently used to study long-term trends in genetic diversity in N. American monarchs [12]. By evaluating the genome of monarch specimens across the breeding range, the authors concluded that the monarch population underwent significant growth approximately 200 years ago, which corresponds with the timing of the clearing of forests in the east by European settlers. In fact, the late Lincoln Brower had even speculated that this had happened [13], though without the use of genetic tools. The Boyle et al. [12] study also reported that there was no evidence of monarch population decline in the past 75 years, nor was there evidence of widespread declines of common milkweed, the most abundant species of milkweed used by monarchs. This evidence paints a picture of a species that is not in trouble, yet this study was not mentioned in the Oberhauser review.

## 3. The MLMP Dataset

**Figure 1:**
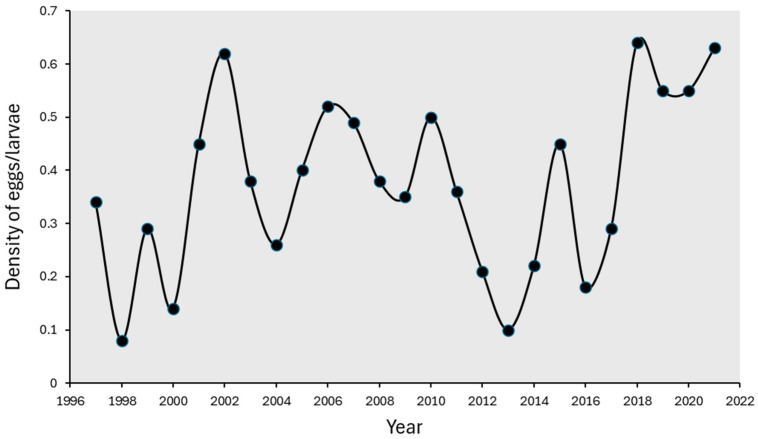
Plot of annual mean number of monarch eggs and larvae per plant across the American Midwest, based on data from the Monarch Larvae Monitoring Project. The data was obtained from supplemental material provided in Pleasants et al. [15]. Each point represents the peak density of the last generation of the summer.

Other important evidence that was noticeably missing from the review was, ironically, data that came from a citizen science program that the author originally spearheaded; the Monarch Larvae Monitoring Project (MLMP), which began in the mid-1990s. This program asks volunteers to survey milkweed patches and report how many monarch eggs, larvae or pupae they see on a weekly basis throughout the summer. Those data have been used by the author (and colleagues) in the past to assess long-term trends in the reproductive output of the population [14] and at that time, it looked like there were population-wide declines in reproductive success. Moreover, Oberhauser even cites this early paper as evidence of “population declines” in the review. However, more recent data from this program, which was included as supporting information in a recent study [15], no longer shows evidence of long-term population declines (see Figure 1 of this critique). Given the author’s close association with this program, and the relevance of the dataset to this topic of population status, the omission of this up-to-date evidence in the review article is puzzling. Importantly though, this evidence also supports the view that the monarch (breeding) population is not in jeopardy.

## 4. Migration Mortality—Tagging Study

The apparent disconnect between the stable summertime monarch abundance, while winter colony sizes have declined, has led to an alternative and still very plausible hypothesis that there has been increasing mortality during the fall migration (the “migration mortality” hypothesis) [16,17]. Oberhauser brings up this explanation but dismisses it based on a single study of monarch tagging data, in which the analyses appeared to show no evidence that tag-recovery rates in Mexico have changed over time [18]. However, Oberhauser fails to point out that the tagging study itself was later rebutted; Fordyce et al. [19] showed that the study was overly reliant on unstandardized tag search efforts, and had also used improper statistics. In fact, based on their own analyses, there seems to have been an increase in tag recoveries in Mexico, which could be interpreted as increased search effort, or increased mortality during the winter. Either way, this study did not refute the migration mortality hypothesis, as Oberhauser suggests.

Finally, an even more recent study also addressed the migration mortality issue by analyzing sightings of migratory roosts, and showed how these roost sizes have been getting smaller at the lower latitudes of the journey [20]. This is consistent with migration mortality, or at least “dropout,” and since this is increasing over time, it would lead to lower winter colony sizes, despite the robust breeding population. Thus, the migration mortality hypothesis remains an entirely plausible explanation for the discordant breeding and winter season trajectories. Though, even if this explanation holds true, the mortality experienced during migration does not justify efforts to bolster breeding season habitat, as the Oberhauser review concludes. And heightened migration mortality, while tragic, does not signify a “declining population.”

## 5. Concluding Remarks

Any review paper should strive to objectively assemble and synthesize all available scientific evidence, yet the Oberhauser [7] article fell short of this goal in multiple instances, thereby painting a misleading picture of the status of monarchs in eastern North America. In this critique, at least three pieces of evidence have been highlighted that were either missing from the review or mischaracterized, including the stable number of breeding adults in the population, the stable densities of eggs and larvae in the Midwest, and the stable levels of genetic diversity. Each of these findings would be welcome news for any species of conservation concern. To dismiss or omit these findings in the ongoing discussion about monarch butterfly conservation matters is disingenuous.

## 6. Response to the Oberhauser and Pleasants Rebuttal

As is customary, the journal editors had provided the original author a chance to respond to this critique. After reading that response, I point out the following:

First, I welcome the comments on each of my concerns above, where I pointed out how certain pieces of evidence or data were visibly missing (or mischaracterized) from the Oberhauser review. In their responses, Oberhauser and Pleasants laid out their interpretation of these studies, and this in itself made it clear that there are differing interpretations of these studies. This discourse is important for readers to see. Not only did this accomplish my goal of highlighting the missing or mischaracterized studies in the original review, but it also made clear that the idea that the “monarchs are declining” depends on which datasets are considered and how they are interpreted.

Second, nowhere in the Oberhauser and Pleasants rebuttal was there an answer to the same running question I posed throughout my critique, which was why was this evidence of a stable population (or the discussion of it) not included in the original review. Why did it take this critique to force the author(s) to recognize and discuss the genomic evidence of population stability [12], for example? Similarly, why was the up-to-date MLMP data from the Midwest region not included in the original review, especially since the author had clearly considered earlier versions of those data relevant to the issue of monarch population trends in 2015 [14]? And why did the author not mention the rebuttal to the Taylor et al. [18] migration mortality study [19] until it was brought up here? As objective scientists, we should be openly discussing the complete body of science around this issue, including the strengths and weaknesses of each study. Omitting or dismissing studies that do not fit a narrative is not objective.
